# Integrated Mitochondrial Genome and Transcriptomic Analyses Reveal Long Non-Coding RNAs Associated with Drought Tolerance in *Sophora moorcroftiana*

**DOI:** 10.3390/biology14121711

**Published:** 2025-11-30

**Authors:** Jun Xu, Yan Sun, Yuting Wang, Jibin Nan, Quzhen Gesang, Bingzhang Li

**Affiliations:** 1 School of Horticulture and Landscape, Yangzhou University, Yangzhou 225009, China; 2 Tibet Academy of Forest Trees, Lasa 851400, China

**Keywords:** *Sophora moorcroftiana*, drought tolerance, mitogenome, RNA editing, transcriptome

## Abstract

*Sophora moorcroftiana* is a perennial deciduous dwarf shrub that exhibits remarkable ecological adaptability, including strong drought resistance on the Tibetan Plateau. In this study, the complete mitogenome of *S. moorcroftiana* was reported and assembled for the first time, representing a circular molecule of 534,205 bp with a GC content of 44.93%. The mitogenome was annotated to include 33 unique protein-coding genes (PCGs), 19 tRNA genes, and three rRNA genes. Phylogenetic and collinearity analyses of the mitogenomes of *S. moorcroftiana* and related species revealed their evolutionary relationships and a non-conserved structure. The codon usage of the PCGs and 166 simple sequence repeats was also analyzed. Conjoint analysis of the transcriptome and mitogenome identified 587 RNA editing sites across 33 PCGs, with 14 genes significantly induced in the roots under drought treatment. Moreover, the levels of proline, soluble sugar, soluble protein, and peroxidase activity were significantly elevated in *S. moorcroftiana* roots subjected to different PEG6000 concentrations. These findings provide valuable insights into the molecular mechanisms underlying drought responses and offer genetic resources for improving drought resistance in *S. moorcroftiana*.

## Simple Summary

Plant mitochondria play essential roles in cellular energy metabolism and have variable and complex structures of their mitogenomes. However, no complete mitogenome has been used to explore the genetic mechanisms underlying drought resistance in *S*. *moorcroftiana*. Here, we sequenced the first mitogenome of *S*. *moorcroftiana* and analyzed its structure, codon usage, evolutionary relationships, and RNA editing site distribution. Conjoint analysis of the mitogenome and transcriptome further identified RNA editing sites and differentially expressed genes, as well as elevated physiological indicators in *S*. *moorcroftiana* roots under drought treatment. The present study offers valuable resources for future studies on the molecular mechanisms and breeding strategies aimed at enhancing drought resistance in this species.

## 1. Introduction

*Sophora moorcroftiana* (Benth.) Baker belongs to the genus *Sophora* and is a perennial deciduous dwarf shrub widely distributed on the Tibetan Plateau [1]. *S. moorcroftiana* exhibits strong ecological adaptability, including high resistance to drought, wind, and sand, as well as tolerance to barren conditions and the presence of medicinal compounds. These traits, especially drought resistance, make the species highly valuable for ecological restoration and economic development in the plateau region [2,3,4]. Drought stress severely affects plant biomass production and physiological, biochemical, morphological, and molecular attributes, with adverse effects on photosynthetic capacity [5]. Approaches for enhancing drought resistance include breeding strategies, changes in physiological and biochemical traits, and molecular and genomic perspectives, including multi-omics technologies [5,6]. However, the genetic mechanisms underlying drought resistance in *S. moorcroftiana* remain largely unidentified.

Previous studies have revealed that photosynthetic efficiency and antioxidative capacity of *S. moorcroftiana* are closely associated with water stress [7]. Transcriptome analysis identified a drought-induced DREB transcription factor gene (*SmDREB1*), which enhanced drought resistance in the model plant *Arabidopsis* through genetic engineering [8]. Furthermore, the assembled genome of *S. moorcroftiana* (737.35 Mb) was analyzed through metabolomic and transcriptomic profiling, leading to the identification of α-amylase and β-fructofuranosidase in sucrose metabolism, which are closely associated with drought adaptation [9,10].

Mitochondria and RNA editing processes play critical roles in modulating osmotic potential and maintaining reactive oxygen species (ROS) homeostasis to regulate drought tolerance [10,11,12]. Plant mitochondria are primary energy suppliers and are essential for cellular energy metabolism [13,14]. They regulate oxidative phosphorylation to generate adenosine triphosphate (ATP) and produce metabolic intermediates required for plant growth, development, and stress resistance [15]. The mitochondrial genome exhibits structural diversity and complexity across species [16,17,18]. RNA editing contributes to nucleotide substitutions, deletions, and insertions, mainly within coding regions, thereby promoting mRNA translatability and altering RNA structure, splicing, and stability [16,19,20]. For instance, the pentatricopeptide repeat protein OsPPR674 regulates RNA editing of the *ccmC* gene, controlling cytochrome c synthesis and influencing rice growth and drought sensitivity [21]. In addition, transgenic maize overexpressing *ZmCYB5-1* (cytochrome b5 protein gene) exhibited significantly reduced drought tolerance compared with wild-type plants [22]. However, the mitochondrial genome (mitogenome or mtDNA) of *S. moorcroftiana* has not been reported, which has restricted genetic studies on the molecular mechanisms of this species.

In this study, a combination of Illumina and Nanopore sequencing data was employed to assemble and annotate the *S. moorcroftiana* mitogenome. Subsequently, the repeat sequences, evolution, DNA transfer from chloroplasts, RNA editing, and genomic recombination events of the *S. moorcroftiana* mitogenome were analyzed. Drought-responsive genes modulated by RNA editing of mitochondrial transcripts were identified through a conjoint analysis of the mitogenome and long non-coding RNA (lncRNA) transcriptome. Physiological indicators, including proline (Pro), malondialdehyde (MDA), soluble sugar, soluble protein, and peroxidase (POD) activity, were also measured in *S. moorcroftiana* seedlings under drought treatment. These results imply that RNA editing events of mitochondrial genes, lncRNA expression, and physiological traits may be related to the drought resistance mechanism of *S. moorcroftiana*, thereby providing genetic resources for future breeding improvement of drought resistance.

## 2. Materials and Methods

### 2.1. Plant Materials, DNA Isolation, and DNA Sequencing

The leaves of two-year-old *S. moorcroftiana* were collected from the Tibet Academy of Forest Trees (N: 29°34′17.92″, E: 91°1′28.73″) in the summer of 2024, and high-quality genomic DNA was isolated using an optimized extraction method [23]. The quality, purity, and concentration of the DNA were determined using a Qubit fluorometer and a NanoDrop spectrophotometer (Thermo Scientific, Waltham, MA, USA). DNA integrity was confirmed by visualization on a 1% agarose gel. Long and short reads were generated using the PromethION sequencer (Oxford Nanopore Technologies, Oxford, UK) and Illumina NovaSeq platform (Illumina, San Diego, CA, USA), respectively, to obtain full-length mitogenome sequences.

### 2.2. Assembly and Annotation of the S. moorcroftiana Mitogenome

The *S. moorcroftiana* mitogenome was assembled using Flye software with the parameters “--min-overlap 2000” based on long-read data to obtain the graphical assembly results in GFA format [24]. Subsequently, for all assembled contigs in FASTA format, the makeblastdb and BLASTn programs (NCBI, Bethesda, MD, USA) were used to build a database and identify contig fragments containing the mitochondrial genome by referencing *Arabidopsis thaliana* sequences, with the parameters set as “-evalue 1e-5 -fmt 6-max_hsps 10-word_size 7-task blastn-short”. Bandage software (v0.8.1) (https://github.com/rrwick/Bandage, accessed on 10 November 2025) was used to generate a draft mitogenome by screening the mitochondrial contigs based on BLASTn results [25]. Subsequently, Unicycler (GPLv3) (github.com/rrwick/Unicycler, accessed on 10 November 2025) and Bandage were used to assemble and visualize the complete *S. moorcroftiana* mitogenome [25,26]. The sequence of the *S. moorcroftiana* mitogenome was submitted to the NCBI under accession number PX569965.

The annotation of the *S. moorcroftiana* mitogenome was performed using reference mitogenomes, including *Sophora japonica* (MG757109), *Medicago truncatula* (KT971339), *M. pinnata* (JN872550), *Vigna angularis* (AP012599), *V. radiata* (HM367685), and *G. max* (JX463295). The PMGA tool (http://1kmpg.cn/pmga/, accessed on 10 November 2025) was used for annotation, with an emphasis on trans-splicing genes and splice sites [27]. tRNA genes were identified using tRNAscan-SE (v.2.0.11) (http://lowelab./tRNAscan-SE/index.html, accessed on 10 November 2025), and rRNA genes were annotated using BLASTn [28]. Finally, Apollo software (v1.11.8) (http://www.ensembl.org/apollo, accessed on 10 November 2025) was used to manually correct errors in the mitogenome annotations [29].

### 2.3. Detection of Repeats and Codon Usage Analysis

The software MISA (v2.1) (https://webblast.ipk-gatersleben.de/misa/, accessed on 10 November 2025), Tandem Repeats Finder (v4.09) (https://tandem.bu.edu/trf/trf.unix.help.html, accessed on 10 November 2025), and the REPuter network server (https://bibiserv.cebitec.uni-bielefeld.de/reputer/, accessed on 10 November 2025) were employed to predict and identify the repeat sequences of the mitogenome, including tandem repeats, dispersed repetitive sequences, and simple sequence repeats (SSRs) [30,31,32]. The Circos package (v0.69.9) (http://circos.ca/in_literature/scientific/, accessed on 10 November 2025) and Excel (2021) were used for result visualization [33]. The protein-coding genes (PCGs) of the *S. moorcroftiana* mitogenome were identified using Phylosuite (v1.1.16) (https://github.com/dongzhang0725/PhyloSuite, accessed on 20 November 2025) and Geneious Prime 2021 [34,35]. Codon usage was analyzed using MEGA (v7.0) (www.megasoftware.net, accessed on 20 November 2025), and the values of the Relative Synonymous Codon Usage (RSCU) were calculated across the 33 PCGs [36].

### 2.4. Phylogenetic Analysis

PhyloSuite (v1.1.16) (https://github.com/dongzhang0725/PhyloSuite, accessed on 20 November 2025) was used to extract and analyze common genes from the mitogenomes of species closely related to *S. moorcroftiana* [34]. Sequence alignment was performed using MAFFT (v7.505) (https://mafft.cbrc.jp/alignment/software/index.html, accessed on 20 November 2025) [37]. IQ-TREE software (v1.6.12) (http://www.cibiv.at/software/iqtree, accessed on 20 November 2025) was subsequently used to construct a phylogenetic tree with the best-fit model (GTR + F + R3), and the bootstrap analysis was assessed with 1000 replicates [38]. Finally, the phylogenetic results were visualized using the iTOL (v4) (https://itol.embl.de/, accessed on 20 November 2025) [39].

### 2.5. Transfer Fragment and Colinear Analysis

A circular *S. moorcroftiana* chloroplast genome was assembled using GetOrganelle software (https://github.com/Kinggerm/GetOrganelle, accessed on 20 November 2025) with the parameters “-R 15-F embplant_pt” [40]. The CPGAVAS2 tool (https://houseandacres.com/cpgavas2, accessed on 20 November 2025) was used to annotate the chloroplast genome [41], and tRNAs were annotated using tRNAscan-SE (v.2.0.11) (http://lowelab./tRNAscan-SE/index.html, accessed on 20 November 2025). rRNA genes were annotated using BLASTn [28]. Subsequently, transfer and homologous fragments were analyzed using the Circos package (v0.69.9) (http://circos.ca/in_literature/scientific/, accessed on 20 November 2025) and BLASTn [33].

Conserved homologous sequences of chloroplast and mitochondrial genomes were identified using BLASTn, and contiguous blocks longer than 500 bp were selected for further analysis. MCScanX software (http://chibba.pgml.uga.edu/mcscan2/, accessed on 20 November 2025) was then employed to construct a multiple synteny plot and assess the sequence similarity between *S. moorcroftiana* and its related species through pairwise alignments [42].

### 2.6. Identification and Distribution Analysis of RNA Editing Sites

Deepred-mt (https://github.com/aedera/deepredmt, accessed on 20 November 2025) was used to predict and analyze RNA editing sites in the mitochondrial PCGs of *S. moorcroftiana* using a convolutional neural network model [43]. All mitochondrial PCGs of *S. moorcroftiana* were extracted and analyzed with probability values > 0.9.

Specifically, the identification requirements for RNA editing sites included: (i) the coverage depth should be at least 100×; (ii) the number of bases with RNA editing should account for more than 10% of the total bases at that site; and (iii) only C-to-U RNA editing types were retained. Based on the transcriptomic data, we first obtained transcripts from the mitochondrial genome by filtering and mapped them to the complete mitochondrial DNA sequences. Subsequently, the differences between DNA and RNA sequences were further compared using Deepred-mt and REDItools (v2.0) (http://code.google.com/p/reditools/, accessed on 20 November 2025) to identify and visualize the distribution of potential RNA editing sites (such as C to U editing) supported by most reads [43,44].

### 2.7. Drought Treatments and Long Non-Coding RNA Transcriptome Assay

Two-year-old *S. moorcroftiana* seedlings were treated with 10%, 20%, and 30% polyethylene glycol (PEG)-6000 solutions using the root-dipping method for 20 d, while an equal volume of distilled water was used as the control. Every 4 d, 100 mL of distilled water or PEG6000 solution was applied to each seedling. Root samples were collected after 20 d of treatment, with three biological replicates per group. Twelve samples were used to construct lncRNA-seq libraries with rRNA removed using the Ribo-off rRNA depletion kit (Vazyme, Nanjing, China). The lncRNA and mRNA sequencing were performed using an Illumina HiSeq 4000 platform (Illumina, Inc., USA). The sequence quality was assessed using FastQC, and reads with low quality, undetermined bases, or adaptor contamination were removed [45]. Clean data were then aligned with the mitogenome of *S. moorcroftiana* using StringTie (v2.2.1) (http://ccb.jhu.edu/software/stringtie/, accessed on 20 November 2025) [46], and mapped to the PCGs and transcripts using Bowtie2 (http://bowtie-bio.sourceforge.net/bowtie2/index.shtml, accessed on 20 November 2025) and TopHat2 (http://ccb.jhu.edu/software/tophat, accessed on 20 November 2025) [47,48]. LncRNAs were a class of RNA molecules with a transcript length of more than 200 nt, which did not encode proteins. The lncRNAs were identified using CNCI (http://www.bioinfo.org/software/cnci, accessed on 20 November 2025) [49], CPC2 (http://cpc2.cbi.pku.edu.cn, accessed on 20 November 2025) [50], and PLEK (https://sourceforge.net/projects/plek/files/, accessed on 20 November 2025) [51], and selected the intersection of these transcripts with no coding potential as a reliable prediction result. The expression levels of lncRNAs between different groups were quantified using StringTie (v2.2.1) (http://ccb.jhu.edu/software/stringtie/, accessed on 20 November 2025) by calculating fragments per kilobase of transcript per million mapped reads (FPKM) values [46,52]. There were three main ways, namely antisense analysis, prediction of cis-acting target genes, and prediction of trans-acting target genes, to predict the lncRNA target genes. We used the ViennaRNA (v2.3.3) (http://www.tbi.univie.ac.at/RNA, accessed on 20 November 2025) and RNAplex (http://www.tbi.univie.ac.at/~htafer/, accessed on 20 November 2025) to predict the base pairing relationship and interaction between antisense lncRNA and mRNA according to the minimum free energy of thermodynamic structure [53,54]. Prediction of cis-acting target genes mainly involved the assumption that lncRNAs located upstream or downstream might participate in gene co-expression, so we selected the target genes within 100 kb upstream and downstream of the lncRNAs. For the prediction of trans-acting target genes, we used the Pearson correlation coefficient method to analyze the correlation between LncRNA and target genes, and the genes with a coefficient greater than 0.9 with LncRNA were selected as their target genes. Differential expression analysis was performed by comparing the expression profiles of lncRNAs, and differentially expressed genes (DEGs) were identified using a threshold of |log_2_ FoldChange| ≥ 0.8 [55]. The Tbtools package (https://github.com/CJ-Chen/TBtools/releases, accessed on 20 November 2025) was used to generate heatmaps to visualize the relative expression levels of lncRNA target genes in the *S. moorcroftiana* mitogenome [56].

### 2.8. Measurement of Various Physiological Indices of S. moorcroftiana Seedlings Under Drought Stress

Detection kits and a spectrophotometer were used to determine the contents of soluble protein, Pro, soluble sugar, and MDA, as well as POD activity in the roots of *S. moorcroftiana* seedlings subjected to drought stress induced by PEG6000 treatment. Distilled water served as the control, and each treatment was performed in three biological replicates.

For MDA quantification, an MDA content assay kit (Order No. D799561, Sangon Biotech, Shanghai, China) and a colorimetric method were employed. According to the manufacturer’s instructions, 0.1 g of the sample was homogenized in 1 mL of extraction solution using a grinder at 0 °C and centrifuged at 4000 rpm for 3 min. The resulting supernatant was centrifuged at 800× *g* at 4 °C for 10 min, and 1.2 mL of the preheated working solution was combined with 0.4 mL of the supernatant and 0.4 mL of reagent three, followed by incubation in boiling water for 1 h. After cooling, the mixture was centrifuged at 10,000× *g* at 25 °C for 10 min, and 1 mL of the supernatant was collected for absorbance measurement at 532 and 600 nm using a spectrophotometer.

Similarly, a Pro content assay kit (Order No. D799575, Sangon Biotech, Shanghai, China) and a POD activity assay kit (Order No. D799591, Sangon Biotech, Shanghai, China) were employed to determine Pro content and POD activity, respectively. The contents of soluble protein and soluble sugar in the roots of *S. moorcroftiana* under drought stress were measured using Coomassie Brilliant Blue and colorimetric methods [57,58]. All experiments were conducted in at least three replicates. GraphPad Prism version 6 was used for statistical analyses, and significant differences between the treatment and control groups were determined using Student’s *t*-test.

### 2.9. Statistical Analysis

The raw sequence data of the *S. moorcroftiana* mitogenome and transcriptome were deposited in NCBI under BioProject accession numbers PRJNA1336633, PRJNA1335579, and PRJNA1335384, respectively. All experimental data were analyzed using the general linear model procedure (SPSS, Ver. 16.0), and analysis of variance (ANOVA) was performed to reveal and assess significant differences among the groups.

## 3. Results

### 3.1. Mitogenome Assembly and Characterization

**Figure 1:**
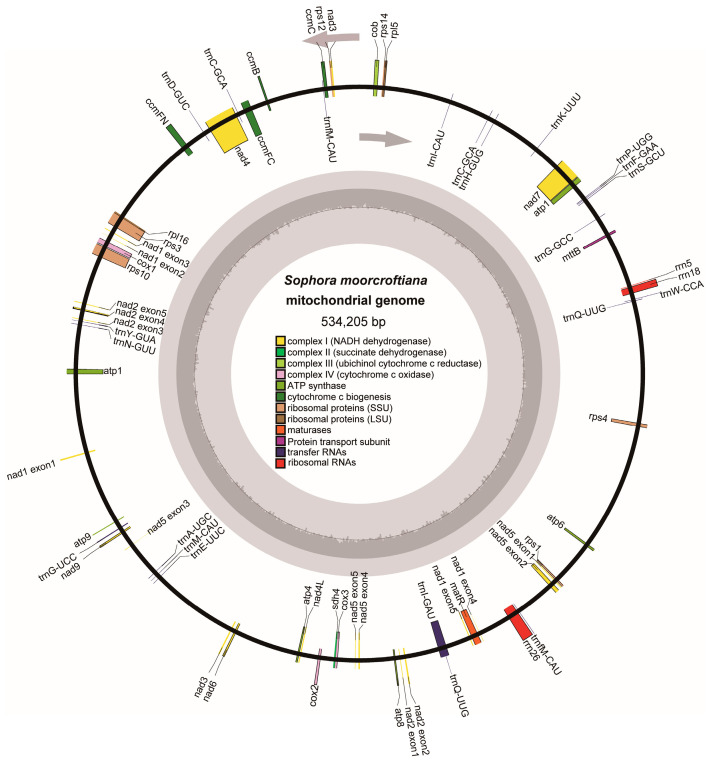
Circular structure of the *S. moorcroftiana* mitochondrial genome. The map illustrates 33 annotated genes shown in different colors, according to their functional classification. Arrows indicate that genes listing outside the circle are transcribed in a clockwise direction, and genes inside are transcribed in a counterclockwise direction.

An accurate mitogenome of *S. moorcroftiana* was successfully assembled and annotated using Illumina and Nanopore sequencing data. The mitogenome consisted of a single circular molecule of 534,205 bp with a GC content of 44.93% (Figure 1 and Table 1). Mitogenome annotation revealed 33 unique PCGs, including two multi-copy genes, with 24 core genes and 9 variable genes. In addition, 19 tRNA genes (three multi-copy) and 3 rRNA genes were annotated (Supplementary Table S1). The core genes were conserved, and functional categorization included nine NADH dehydrogenase genes, five ATP synthase genes, four cytochrome *c* biogenesis genes, three cytochrome *c* oxidase genes, one ubiquinol–cytochrome *c* reductase gene, one membrane transport protein gene, and one maturation enzyme gene. The nine variable genes included two ribosomal protein large subunit genes, six ribosomal protein small subunit genes, and one succinate dehydrogenase gene (Figure 1 and Supplementary Table S1).

### 3.2. Repeat Sequences and DNA Transfer Analysis

**Figure 2:**
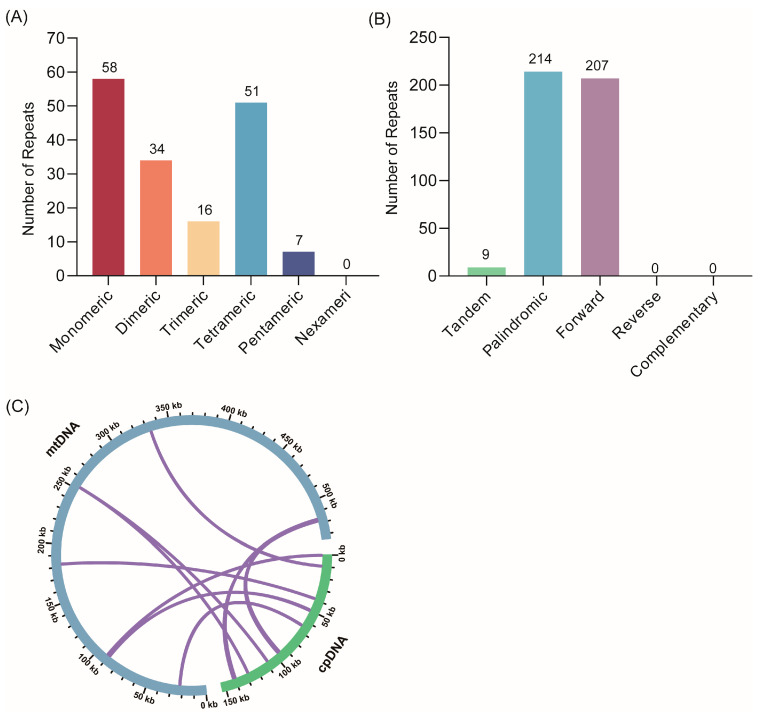
Repeat elements and cpDNA transfer in the *S. moorcroftiana* mitogenome: (**A**) Number and types of SSRs distributed in the mitogenome. (**B**) Numbers and types of tandem and dispersed repeats. The x-axis indicates the repeat types, and the y-axis indicates the number of repeated fragments. (**C**) DNA sequence migration analysis. The green and blue arcs indicate the chloroplast genome and mitogenome, respectively. The purple line connecting the arcs corresponds to homologous genomic segments.

The mitogenome contains tandem and dispersed repeats that are frequently used as molecular markers to examine host phylogeny. To investigate these repeat fragments in the *S. moorcroftiana* mitogenome, we analyzed the distribution of SSRs, tandem repeats, and dispersed repeats. A total of 166 SSRs were identified, including 58 monomers, 34 dimers, 16 trimers, 51 tetramers, and 7 pentamers (Figure 2A and Supplementary Table S2). Nine tandem repeats with similarities above 81% and lengths ranging from 12 to 28 bp were also detected (Supplementary Table S3). Moreover, 421 dispersed repeats were observed, with lengths ≥ 30 bp, comprising 214 palindromic and 207 forward repeats. The longest palindromic repeat was 308 bp, and the longest forward repeat was 2755 bp. No reverse or complementary repeats were identified in the *S. moorcroftiana* mitogenome (Figure 2B and Supplementary Table S4).

Furthermore, chloroplast fragments integrated into the *S. moorcroftiana* mitogenome were examined. Sequence similarity and length of the migrating fragments varied among species, and seven homologous fragments were identified between the chloroplast and mitochondrial genomes of *S. moorcroftiana*. These fragments totaled 2816 bp, representing approximately 0.53% of the mitogenome (Figure 2C). The longest fragment, MTP1, was 1617 bp, and four additional tRNA genes (*trnW*-CCA, *trnN*-GUU, *trnH*-GUG, and *trnM*-CAU) were detected within these homologous fragments.

### 3.3. Codon Usage Analysis of S. moorcroftiana Mitochondrial Genome

**Figure 3:**
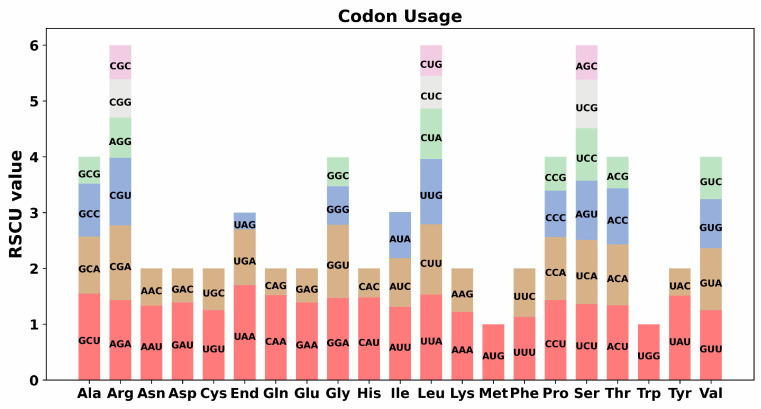
Codon usage preferences in the *S. moorcroftiana* mitochondrial genome. The y-axis indicates the RSCU values, which show the preferences among synonymous codons.

Codon usage bias in the mitochondrial genome represents an important evolutionary phenomenon arising from the interplay of natural selection, gene mutation, and genetic drift. To investigate the codon usage of the *S. moorcroftiana* mitogenome, the codon distribution across 33 PCGs was systematically analyzed, and the corresponding amino acid changes were examined (Supplementary Table S5). Codon usage was observed to be preferentially associated with amino acids having RSCU values > 1. A general preference for codon usage was identified in the *S. moorcroftiana* PCGs, except for the start codon AUG and tryptophan codon UGG. Additionally, a notable preference was observed for the stop codon UAA, which exhibited the highest RSCU value of 1.7 (Figure 3 and Supplementary Table S5).

### 3.4. Phylogenetic and Collinearity Analysis

**Figure 4:**
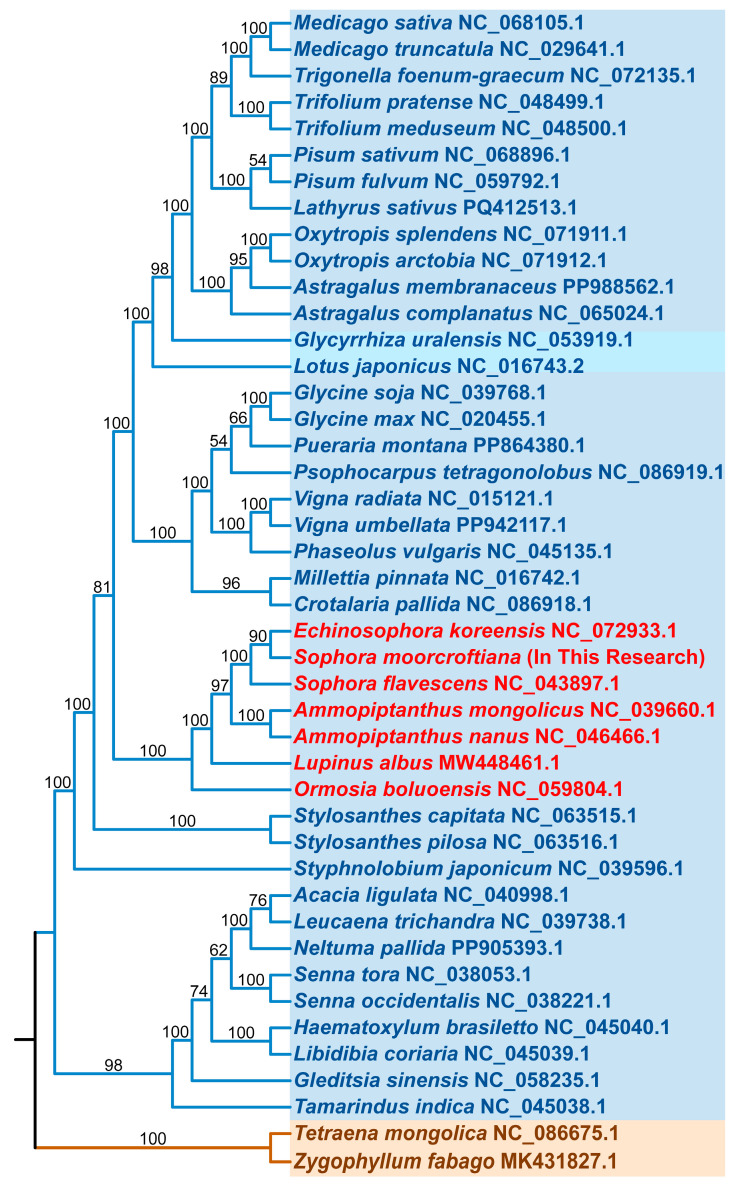
Phylogenetic analysis of *S. moorcroftiana* and related genera. A phylogenetic tree was constructed for the mitogenomes of *S. moorcroftiana* and 43 other plant species using the maximum-likelihood method. Bootstrap support values are shown at the nodes, and branch colors indicate different taxonomic orders.

To further explore the evolutionary relationships of the *S. moorcroftiana* mitogenome, all genes from the mitogenomes were analyzed along with the sequences of 43 closely related species obtained from GenBank. A total of 21 shared PCGs were selected to construct a phylogenetic tree. The results indicated that 42 mitogenomes, including that of *S. moorcroftiana*, clustered within Fabales species, whereas two mitogenomes of Zygophyllales were adopted as the outgroup (Figure 4 and Supplementary Table S6). The mitochondrial DNA-based analysis confirmed the placement of *S. moorcroftiana* within the order Fabales, family Fabaceae. Seven closely related species from the same group, namely *Echinosophora koreensis*, *Sophora flavescens*, *Ammopiptanthus mongolicus*, *A. nanus*, *Lupinus albus*, and *Ormosia boluoensis*, were selected for comparative analysis based on their homology (Figure 4). High-quality mitogenomes of these seven species were obtained from NCBI, with GC content and genome size (Supplementary Table S6).

**Figure 5:**
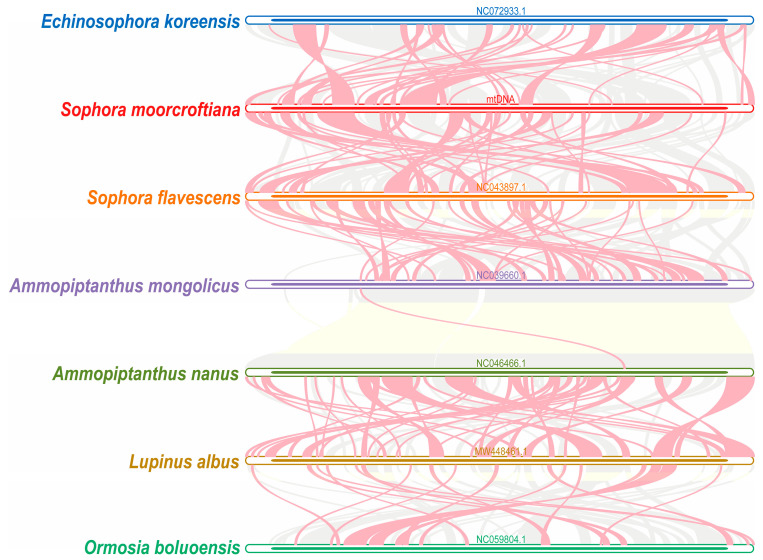
Collinearity analysis between mitogenomes of *S. moorcroftiana* and six related species. Each bar represents a mitogenome, and ribbons denote homologous regions between *S. moorcroftiana* and the other six related plant species. The gray area indicates regions with homology, and the red arc area indicates inverted regions.

To further investigate the homology of the *S. moorcroftiana* mitogenome, collinearity analysis was performed using seven closely related species within Fabaceae. MCScanX was used to construct multiple synteny plots. The results revealed multiple colinear blocks between *S. moorcroftiana* and the six other closely related species. The red arc region represents the inverted regions, whereas the gray region represents the colinear blocks with good synteny (Figure 5). Numerous collinear blocks were identified, whereas blocks shorter than 0.5 kb were excluded. These findings demonstrate that collinearity orders among the seven species were inconsistent and not structurally conserved, suggesting that the *S. moorcroftiana* mitogenome underwent extensive rearrangements compared with those of closely related species.

### 3.5. Characteristics and Distribution of RNA Editing Sites

**Figure 6:**
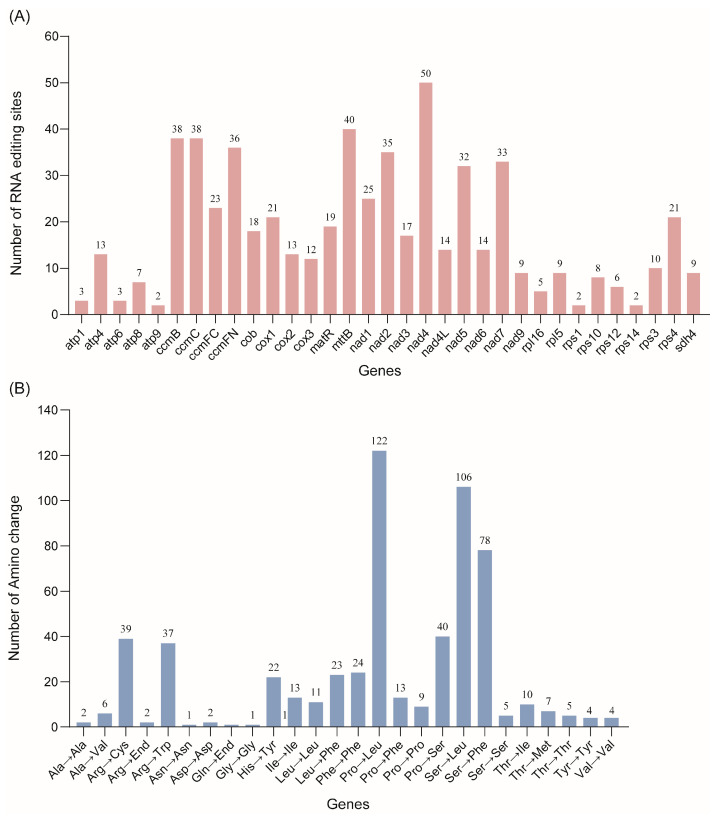
Distribution of RNA editing sites identified in the PCGs of the *S. moorcroftiana* mitogenome: (**A**) Number of RNA editing sites distributed across *S. moorcroftiana* PCGs. The x-axis indicates gene names, and the y-axis indicates the number of RNA editing sites. (**B**) Changes in the encoded amino acids caused by RNA editing. The x-axis represents the type of amino acid change, and the y-axis indicates the number of amino acid changes.

RNA editing events are categorized as a form of post-transcriptional modification that increases the similarity of mitochondrial protein sequences in plant species. This process alters amino acid sequences, frequently converting hydrophilic amino acids into hydrophobic ones, thereby enhancing protein folding and its stability. Moreover, RNA editing generates novel start and stop codons, producing proteins with greater conservation in those species. The *S. moorcroftiana* transcriptome and mitogenome were further analyzed using Deepred-mt and REDItools to identify and visualize the distribution of potential RNA editing sites. A total of 587 C-to-U editing sites were identified across 33 PCGs in the *S. moorcroftiana* mitogenome. Notably, the number of editing sites varied across different genes, with *nad4* and *mttB* genes containing 50 and 40 sites, respectively (Figure 6A and Supplementary Table S7). These editing sites were predominantly located at non-synonymous positions, leading to amino acid substitutions. In addition, 81 potential synonymous mutation sites were identified. The majority of RNA editing resulted in three amino acid substitutions: Pro to Leu (No. 122), Ser to Leu (No. 106), and Ser to Phe (No. 78) (Figure 6B).

### 3.6. Comprehensive Analysis of the Mitogenome and lncRNAs Involved in Drought Tolerance of S. moorcroftiana

**Figure 7:**
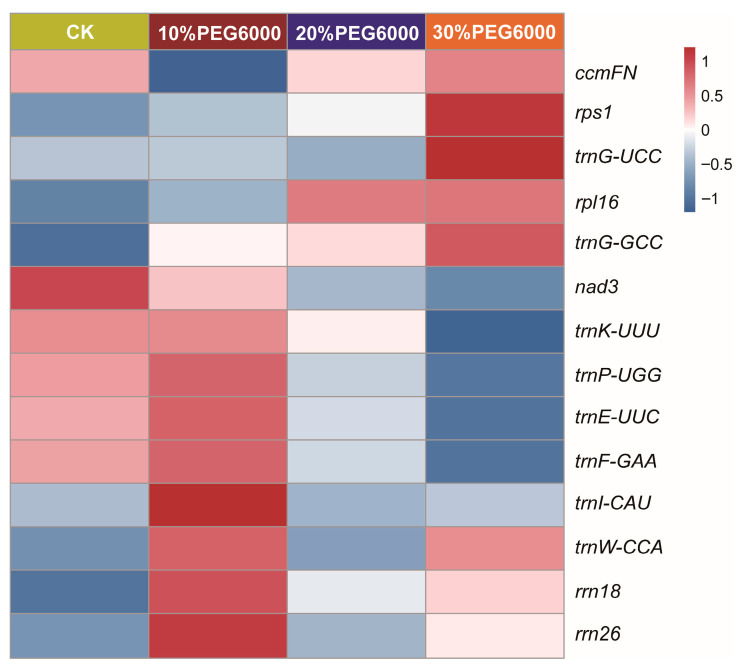
Transcriptomic analysis of lncRNA–target genes interactions related to drought tolerance in *Sophora moorcroftiana*. Heatmap showing the expression of related DEGs in *S. moorcroftiana* roots under 10%, 20%, and 30% (*w*/*v*) PEG6000 treatments. The expression levels were calculated using FPKM data. The color scale represents the values with Z-score normalization. Gene expression differences are shown using color bars, with red and blue indicating the genes that are more highly or weakly expressed, respectively. Horizontal and vertical labels represent the treatments and gene names, respectively.

To further examine drought-responsive genes in the mitogenome, lncRNA and mRNA sequencing were performed to analyze the expression of lncRNAs and their target genes in *S. moorcroftiana* under 10%, 20%, and 30% (*w*/*v*) PEG6000 treatments. LncRNAs primarily regulate target gene expression through three mechanisms: base-pairing with mRNAs, cis-regulation, and trans-regulation. The results revealed that 14 genes were significantly induced by PEG6000 treatment compared with the control, using a threshold of |log_2_ FoldChange| ≥ 0.8. Among them, eight genes (*rrn18*, *rrn26*, *rps1*, *rpl16*, *trnI*-CAU, *trnG*-UCC, *trnG*-GCC, and *trnW*-CCA) were upregulated, and six genes (*ccmFN*, *nad3*, *trnE*-UUC, *trnF*-GAA, *trnK*-UUU, and *trnP*-UGG) were downregulated after PEG6000 treatments (Figure 7). Notably, *rrn18*, *rrn26*, and *trnW-CCA* exhibited high expression levels and were upregulated after 10% and 30% (*w*/*v*) PEG6000 treatments. *rpl16*, *rps1*, *trnG-UCC,* and *trnG-GCC* were significantly upregulated and induced by 30% (*w*/*v*) PEG6000 treatment. Additionally, transcript level of *ccmFN* was downregulated after 10% (*w*/*v*) PEG6000 treatment, whereas *nad3* was significantly downregulated and induced by 20% and 30% (*w*/*v*) PEG6000 treatments. The transcript levels of *trnE-UUC*, *trnF-GAA*, *trnK-UUU*, and *trnP-UGG* were significantly higher and downregulated in the 30% (*w*/*v*) PEG6000-treated seedlings. These observations suggest that the identified genes are closely associated with drought resistance. Functional category analysis indicated that DEGs were enriched in categories such as organic cyclic compound binding, NAD(P)H dehydrogenase activity, ribosomes, non-membrane-bound organelles, and so on (Supplementary Figure S1).

### 3.7. Physiological Responses of S. moorcroftiana Seedlings Under Drought Stress

**Figure 8:**
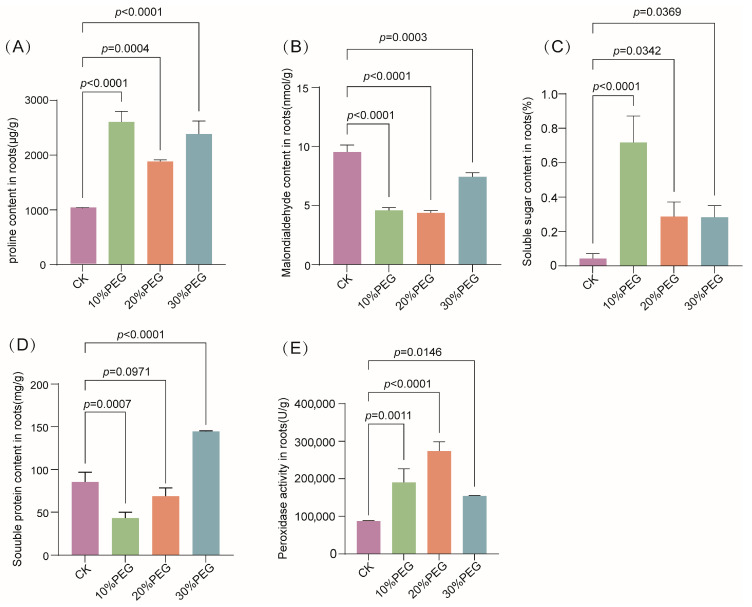
Changes in osmoregulatory substances and peroxidase activity in *S. moorcroftiana* under PEG6000 treatment: (**A**–**D**) Contents of proline (Pro), malondialdehyde (MDA), soluble sugar, and soluble protein in *S. moorcroftiana* roots under 10%, 20%, and 30% (*w*/*v*) PEG6000 treatments. (**E**) Peroxidase (POD) activity in *S. moorcroftiana* roots under the same treatments. Data are presented as mean ± SD (*n* = 3). Significant differences were determined using Dunnett’s multiple comparison test (each treatment vs. each control, *n* = 3).

Pro, MDA, soluble sugars, and soluble proteins are key osmoregulatory substances that contribute to drought resistance by maintaining intracellular osmotic pressure and reducing water loss. Moreover, plants enhance antioxidant activities, such as POD activity, to mitigate cellular damage and thereby strengthen overall drought resistance. Changes in physiological indicators, including Pro, soluble sugar, soluble protein, and MDA, as well as POD activity, were measured in *S. moorcroftiana* seedlings subjected to 10%, 20%, and 30% (*w*/*v*) PEG6000 treatments. The Pro content and POD activity exhibited significant increases following PEG6000 treatment. Similarly, soluble sugar and protein contents increased markedly under 10% and 30% PEG6000 treatments, respectively. Conversely, MDA levels were significantly reduced following PEG6000 treatment (Figure 8). These results indicate that high levels of osmoregulatory substances and enhanced antioxidant enzyme activity are pivotal for the drought resistance of *S. moorcroftiana* seedlings under drought stress.

## 4. Discussion

### 4.1. Characteristic of S. moorcroftiana and Its Mitogenome

*S. moorcroftiana* is an endemic leguminous shrub that occurs exclusively in the arid and semi-arid regions of the Tibetan Plateau, exhibiting high drought and sand tolerance, rendering it valuable for ecological restoration [1,2,3]. This species possesses a deep and extensive root system that ensures its survival in highly drought-prone areas [10]. Previous studies have demonstrated that mitochondria can generate ROS and other metabolites, some of which act as retrograde signals essential for stress responses [59,60]. In the present study, the complete *S. moorcroftiana* mitogenome was analyzed and annotated with 33 unique protein-coding genes, including ATP synthase, cytochrome c biogenesis, and NADH dehydrogenase (Figure 1 and Supplementary Table S1). Mitochondrial dysfunction involving pentatricopeptide repeat proteins or DEXH-box RNA helicases has been reported to regulate ROS accumulation and influence plant adaptation to the stress hormone abscisic acid (ABA) [61]. Similarly, mitochondrial complex I mutants have been found to cause excessive ROS accumulation, suppress the expression of cold-responsive genes, and confer chilling and freezing sensitivities [62]. Mutants or alterations affecting mitochondria have also revealed the importance of maintaining antioxidant balance and organelle interactions to enhance stress resistance [63]. In addition, 166 SSRs and 421 pairs of repeat sequences were identified in the *S. moorcroftiana* mitogenome (Figure 2). These sequences serve as molecular markers and may be valuable genetic resources for future research. Furthermore, phylogenetic tree construction and collinearity analysis between *S. moorcroftiana* and its closely related species revealed high levels of genome rearrangement in the protein-coding genes (Figure 4 and Figure 5). Such structural features of the mitogenome could offer important insights into the molecular mechanisms underlying drought tolerance and may serve as a reference for breeding strategies to enhance drought resistance in *S. moorcroftiana*.

### 4.2. RNA Editing Events in the S. moorcroftiana Mitogenome

Plant mitochondria exhibit numerous RNA editing events characterized by C-to-U nucleotide modifications [64]. RNA editing sites have been identified in both PCGs and non-coding regions, including introns, UTRs, and tRNAs [64,65]. Considerable variations in the number of editing sites have been observed among plant species, with 200–700 sites typically detected in angiosperms and approximately 500 in gymnosperms [66,67]. In the present study, 587 RNA editing sites were identified across the 33 PCGs of the *S. moorcroftiana* mitogenome, all of which represented C-to-U modifications (Figure 6). Notably, *nad4*, *mttB*, *ccmC*, *ccmB*, and *ccmFN* exhibited extensive editing, with more than 35 editing sites each. The tomato (*Solanum lycopersicum*) gene *SlWHY2* encodes a mitochondrial single-stranded DNA-binding protein that regulates the expression of mitochondrial *NAD4*, thereby maintaining mitochondrial function and enhancing drought tolerance [68]. In maize (*Zea mays* L.), the empty pericarp9 (emp9) mutant abolishes the C-to-U editing of *ccmB*-43 and *rps4*-335 sites in mitochondria, thereby affecting the maturation of cytochrome c and impairing the biogenesis of mitochondrial respiratory complexes [69]. These RNA editing sites participate in complex biological processes that regulate plant evolution, development, and adaptation [70,71].

Among these sites, 16 synonymous mutations and 474 non-synonymous changes were identified, altering amino acid sequences. Previous studies have indicated that RNA editing tends to increase amino acid conservation, thereby enhancing protein folding and physicochemical properties to support multiple biological functions [72]. Two stop codons (UGA) and seven conventional start codons (AUG) were detected owing to RNA editing site changes in the *S. moorcroftiana* mitogenome (Supplementary Table S7). Both stop codons resulted from CGA-to-UGA conversions in *ccmFC* and *rps10*, which contributed to truncated amino acid sequences. Nine start codons were introduced by ACG-to-AUG conversions in *rps1*, *rps10*, *cob*, *cox2*, *nad1*, *nad4L*, *nad5*, and *nad7*, thereby enabling translation initiation with methionine. Previous studies have demonstrated that C-to-U RNA editing in the mitogenome increases UV resistance in higher plants [73]. RNA editing can also alter protein translation and play a critical role in plant acclimatization and survival [74,75]. In *Triticum aestivum*, RNA editing patterns differ between drought-tolerant and drought-sensitive cultivars, inducing structural and functional modifications of *NAD9* to enhance drought resilience [76]. Rice pentatricopeptide repeat (PPR) genes are involved in the regulation of mitochondrial RNA editing. PPR035 regulates RNA editing at *rps4*-926 and *orfX*-406, whereas PPR406 regulates RNA editing at *orfX*-355, both contributing to improved drought tolerance in upland rice [77]. Collectively, the identification and analysis of RNA editing sites in the *S. moorcroftiana* mitogenome offer promising prospects for improving drought resistance and provide essential insights into the molecular mechanisms underlying its remarkable drought tolerance.

### 4.3. Plant Mitochondria and the RNA Editing Process Regulate Drought Tolerance

Drought stress is recognized as a major environmental factor that negatively influences plant growth and development by causing excessive water loss, reduced photosynthetic activity, and altered stomatal conductance [78,79]. Mitochondria and the RNA editing process within them play critical roles in regulating intracellular ATP production and maintaining ROS homeostasis, thereby contributing to plant adaptation to drought stress and growth [21,80,81]. Through the conjoint analysis of the *S. moorcroftiana* mitochondrial genome and transcriptome, eight genes were identified as upregulated (*rrn18*, *rrn26*, *rps1*, *rpl16*, *trnI-*CAU, *trnG-*UCC, *trnG-*GCC, and *trnW-*CCA) and six as downregulated (*ccmFN*, *nad3*, *trnE-UUC*, *trnF-*GAA, *trnK-*UUU, and *trnP-*UGG) under drought treatment (Figure 7). In rice, *OsPPR674* knockout plants exhibited ROS accumulation due to defective RNA editing of the mitochondrial cytochrome *ccmC* gene, resulting in reduced plant height and poor drought tolerance [21]. Furthermore, ABA has been shown to induce H_2_O_2_ accumulation in guard cell mitochondria, promoting stomatal closure, reducing water loss, and ultimately enhancing drought resistance [11]. In cotton, mitochondrial genome and transcriptome analyses revealed 339 RNA editing sites distributed across 28 *GhAGC* genes, with *GhAGC5*, *GhAGC9*, *GhAGC23*, and *GhAGC24* exhibiting high transcript abundance under drought stress [82]. Overall, these DEGs and RNA editing sites in the *S. moorcroftiana* mitogenome were strongly correlated with drought resistance and performed important functions in regulating plant stress tolerance in future studies.

## 5. Conclusions

In this study, the *S. moorcroftiana* mitogenome was sequenced, assembled, and annotated to include 33 unique PCGs, 19 tRNA genes, and three rRNA genes, representing a circular molecule of 534,205 bp, with a GC content of 44.93%. Comparative analyses were performed on genome structure, repeat sequences, codon usage, phylogenetic relationships, and RNA editing site distribution. Furthermore, conjoint analysis of the transcriptome and mitogenome identified 587 RNA editing sites across 33 PCGs, and found that 14 lncRNA–target genes were significantly induced in the roots under drought treatment. In addition, physiological indicators, including Pro, soluble sugar, soluble protein, and peroxidase activity, in *S. moorcroftiana* seedlings were significantly induced by PEG6000 treatment. These findings provide comprehensive insights into the molecular basis of drought tolerance in *S. moorcroftiana*, offering valuable resources for future studies on the molecular mechanisms and breeding strategies aimed at enhancing drought resistance in this species.

## Figures and Tables

**Table 1:** Basic information on the *S. moorcroftiana* mitogenome.

Type	Mitochondrial Genome
Circular molecular number	1
Structure	circular
Total length	534,205 bp
GC content	44.93%
Depth (×)	104

## Data Availability

Our mitochondrial genomic and transcriptomic data associated with this study have been submitted to the NCBI under accession numbers PRJNA1336633, PRJNA1335579, and PRJNA1335384, respectively.

## Supplementary Materials

The following supporting information can be downloaded at: https://www.mdpi.com/article/10.3390/biology14121711/s1, Figure S1: GO enrichment analysis of the DEGs showing the GO terms; Table S1: The encoding genes of *S. moorcroftiana* mitogenome; Table S2: The SSRs in the mitochondrial genome of *S. moorcroftiana*; Table S3: Tandem repeat sequences in the mitochondrial genome of *S. moorcroftiana*; Table S4: Dispersed repeat sequences in the mitochondrial *genome of S. moorcroftiana*; Table S5: Relative synonymous codon usage of each amino acid pair in the *S. moorcroftiana* mitochondrial genome; Table S6: The 44 homology of the mitogenomes; Table S7: The identified RNA editing sites in the *S. moorcroftiana* mitogenome.
