# Supplementary material for: Integrated Mitochondrial Genome and Transcriptomic Analyses Reveal Long Non-Coding RNAs Associated with Drought Tolerance in Sophora moorcroftiana
Source: Biology (Basel). 2025 Nov 30;14(12):1711. doi: 10.3390/biology14121711 (PMC12730227; doi:10.3390/biology14121711)
Supplement: Supplementary file 1 [file biology-14-01711-s001.zip › Supplementary figure in a PDF.pdf]

Integrated mitochondrial genome and transcriptomic analyses reveal long non-coding RNAs associated with drought tolerance in *Sophora moorcroftiana*

Jun Xu[1, 2], Yan Sun[2], Yuting Wang[2], Jibin Nan[2], Quzhen Gesang[2*], Bingzhang Li[2*].
[1]School of Horticulture and Landscape, Yangzhou University, Yangzhou, Jiangsu 225009, China;
[2]Tibet Academy of Forest Trees, Lasa, Xizang 851400, China.

Correspondence:
*Corresponding authors, E-mails: 13908981312@163.com (Bingzhang Li); 18889089020@163.com (Quzhen Gesang).

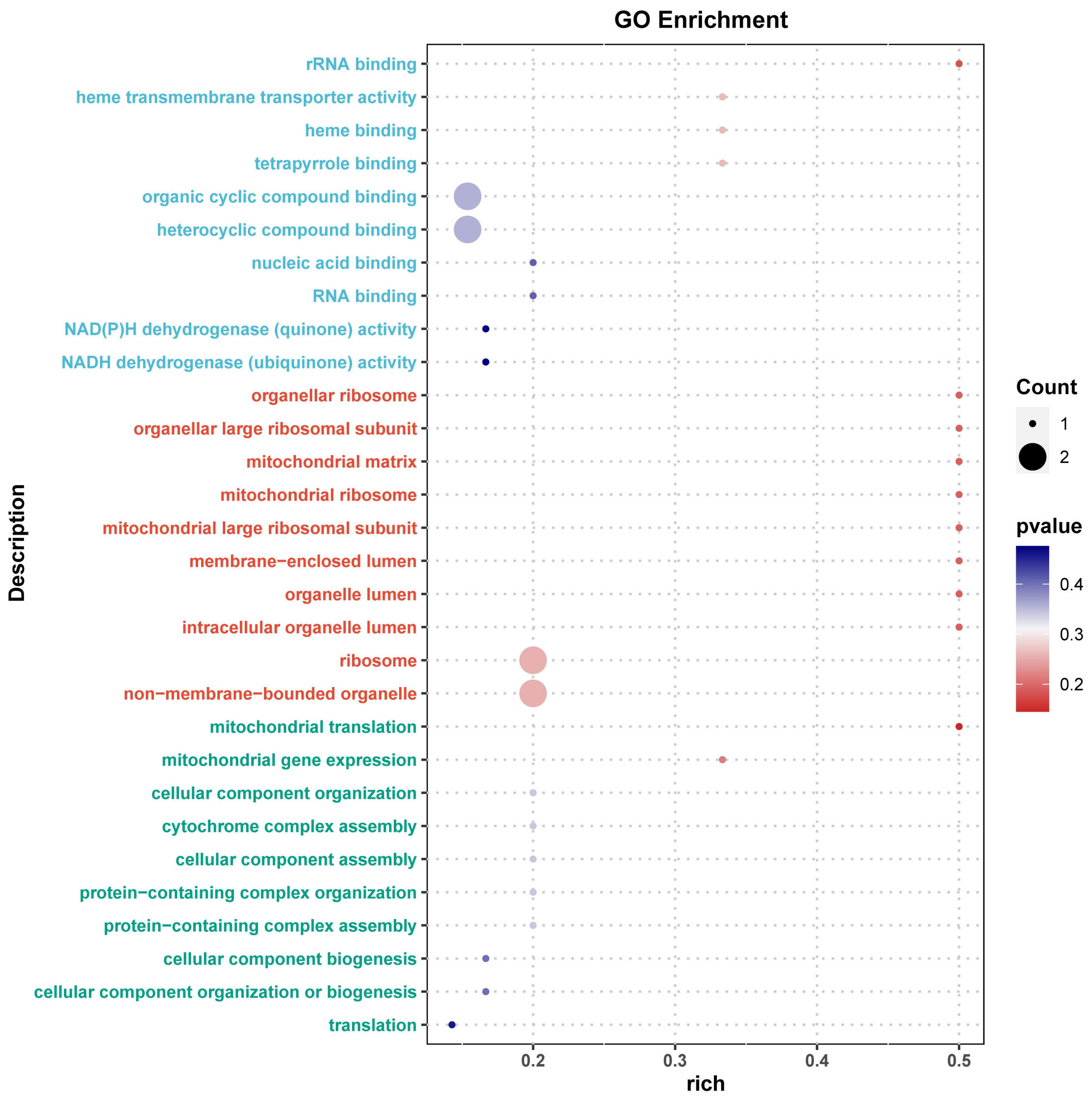

Figure S1 GO enrichment analysis of the DEGs showing the GO terms. Functional category analysis indicated that DEGs were enriched in categories such as organic cyclic compound binding, NAD(P)H dehydrogenase activity, ribosomes, and non-membrane-bound organelles.
