# Supplementary figures and images for: Integrated Mitochondrial Genome and Transcriptomic Analyses Reveal Long Non-Coding RNAs Associated with Drought Tolerance in Sophora moorcroftiana
Source: Biology (Basel). 2025 Nov 30;14(12):1711. doi: 10.3390/biology14121711 (PMC12730227; doi:10.3390/biology14121711)

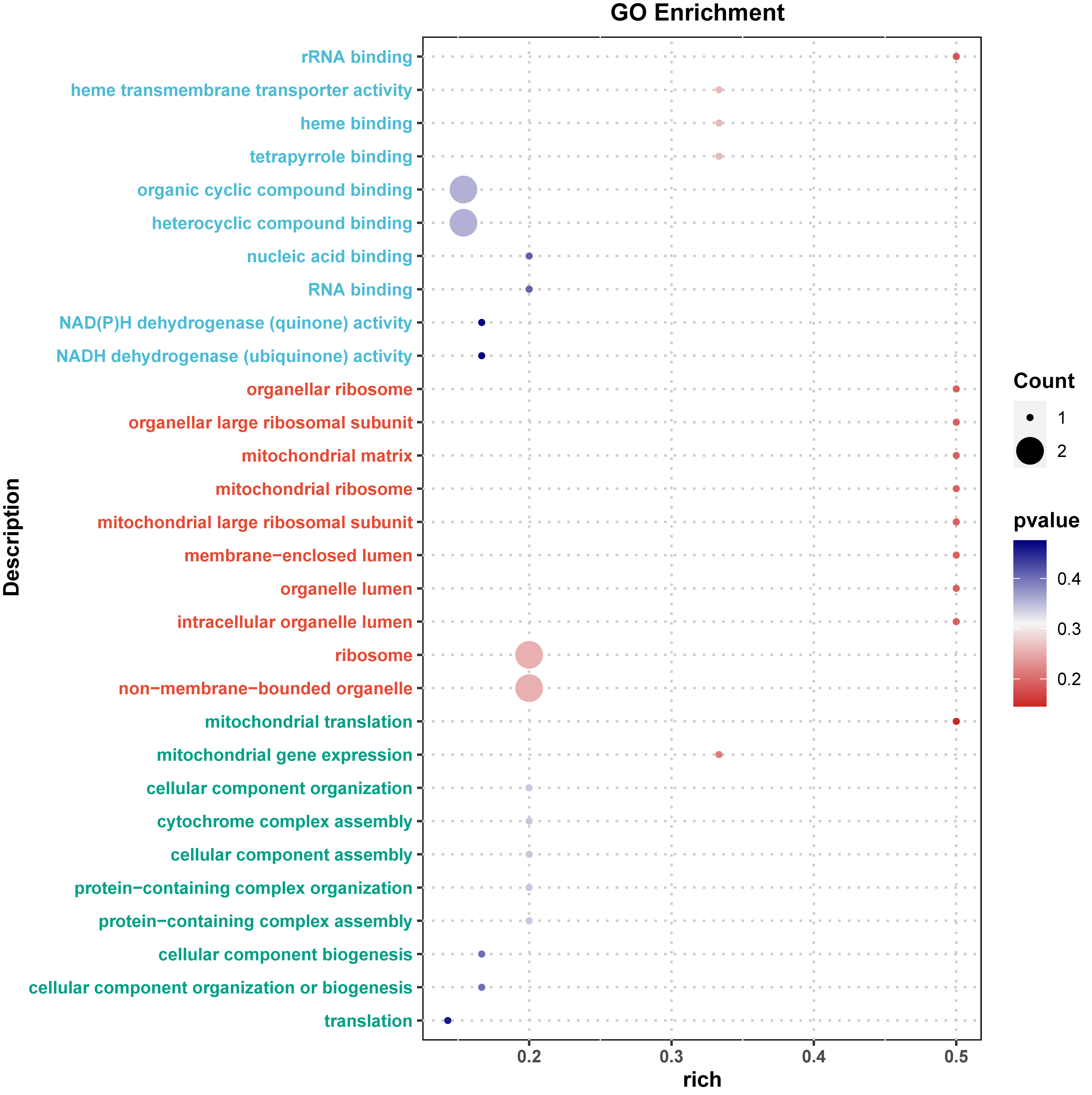

Supplement: Supplementary file 1 [file biology-14-01711-s001.zip › Supplementary figure 1.tif]
